# Gestational Obesity Impairs Maternal Glucose Metabolism in Post-Partum Ewes

**DOI:** 10.3390/ani15243529

**Published:** 2025-12-08

**Authors:** Jason R. McKnight, Michael Carey Satterfield, Fuller W. Bazer

**Affiliations:** Department of Animal Science, Texas A&M University, College Station, TX 77843, USA; jmcknight@tamu.edu (J.R.M.); carey.satterfield@ag.tamu.edu (M.C.S.)

**Keywords:** obesity, insulin resistance, nutrition, pregnancy, glucose metabolism, sheep

## Abstract

This study determined the effects of gestational obesity on the short- and long-term metabolic statuses of post-partum mothers and whether obesity management during pregnancy could attenuate these effects. At 120 days prior to estrus, eight ewes received 100% of the National Research Council (NRC) nutrient requirements (control group), and twenty-four ewes had free access to feed (obesity induction). Embryos from superovulated ewes with normal body condition were transferred to the uteri of control-fed or obese ewes on day 6 post-estrus to generate genetically similar singleton pregnancies. Thirty-five days after embryo transfer, eight obese ewes were subject to restricted feeding (65% of feed intake for the 100% NRC-fed control group) until parturition, and the remaining sixteen obese ewes (eight ewes/group) continued to have free access to feed throughout gestation. Following parturition, all but one group of obese ewes (that continued to have free access to feed) were fed 100% of NRC nutrient requirements. At post-partum days (PPD) 1 and 150, glucose tolerance tests were conducted with ewes. At both PPD1 and PPD150, obesity resulted in insulin resistance, impairment of whole-body glucose utilization, increased levels of circulating leptin, and altered profiles of amino acids in plasma, and these effects were attenuated in ewes receiving obesity management during or after gestation. At PPD150, obesity increased circulating levels of ammonia and urea in ewes, which were prevented by realimentation to 100% NRC requirements. These results indicate that weight reduction in obese ewes during pregnancy or after parturition can ameliorate the adverse metabolic effects of gestational obesity in ewes.

## Simple Summary

Ewes (a well-established animal model for studying human pregnancy) can exhibit an overweight or obesity phenotype in production settings throughout the world. Likewise, these disorders are major global health problems for women of reproductive age. In both species, obesity increases the risks of pregnancy complications. Although there have been extensive studies to determine how gestational obesity affects the development and health of offspring, there is limited research on the metabolic effects of obesity on mothers after parturition. In this study, pre-pregnancy obesity was induced in ewes by ad libitum feeding for 4 months, and beginning on day 35 post-embryo transfer, some obese ewes were subject to restricted feeding (65% of feed intake for the control group) until parturition. Results indicated that maternal obesity during gestation impaired glucose utilization and altered plasma amino acid and fatty acid profiles in ewes on both days 1 and 150 post-partum. These abnormalities were ameliorated by restricted feeding during gestation. Our findings are expected to beneficially guide the nutritional management of obese ewes and women during gestation.

## 1. Introduction

Overweight and obesity in animals result from a chronic imbalance between energy intake (e.g., overeating diets such as manufactured feeds) and energy expenditure (e.g., inadequate physical activity or a sedentary lifestyle) [1,2,3]. These metabolic states can occur in ewes under production conditions in many regions of the world. For example, 5–10% of ewes in breeding seasons are fat or obese based on body condition scores (BCSs) using a scale of 1–5 with 0.5 increments (BSCs of 1.0, 2.0, 3.0, 4.0, 4.5, and 5.0 being emaciated, thin, normal, fat, obese, and very obese, respectively) in the southeastern United States [4], Turkey [5], Scotland [6], Australia [7], and New Zealand [8]. Likewise, overweight or obesity is a major health problem for women of reproductive age worldwide [9]. In both ewes [10,11,12] and women [13,14,15], obesity increases the risks of infertility, metabolic syndrome, and congenital abnormalities. The best way to avoid these adverse effects of maternal obesity is to prevent excessive weight gain before pregnancy. However, this is often not practical as many women enter pregnancy unknowingly, thereby necessitating treatment and action during gestation to minimize obesity-associated complications [16].

Interest in the field of epigenetics, fetal programming, and the developmental origins of health and disease hypothesis [17] have led to numerous studies to define how gestational obesity affects metabolic programming (including insulin resistance) as well as circulating levels of hormones (insulin, cortisol, and leptin) and glucose in the offspring of sheep [18,19,20,21] and humans [14,15,22,23,24,25,26]. In addition, gestational obesity impairs lactation [27], induces insulin resistance [28,29], and increases plasma concentrations of lipids [30] in post-partum mothers. However, there has been limited research on other metabolic effects (e.g., glucose kinetics and amino acid availability) of gestational obesity (without diabetes mellitus) on mothers after parturition in either animals, including ewes [31] or women [32], although maternal health and well-being remain important matters [1,2,3]. We hypothesized that maternal obesity has both short- and long-term impacts on insulin sensitivity in mothers and that reducing obesity during pregnancy or after parturition may ameliorate this metabolic problem. This study only evaluated the interplay between pregnancy and obesity. This hypothesis was tested in the current study using the pregnant ewe (*Ovis aries*), a widely used animal model for research relevant to human pregnancy [11,33]. Results of this study may help develop effective means to improve reproductive health in both sheep and humans.

## 2. Materials and Methods

All surgical and experimental procedures complied with the Guide for the Care and Use of Agricultural Animals in Research and Teaching and were approved by the Institutional Animal Care and Use Committee of Texas A&M University. Prior to the initiation of this study, mature ewes were fed a soybean hull-, wheat middlings-, and corn-based diet (20 g feed/kg body weight per day) [34] to meet requirements for all nutrients recommended by the National Research Council (NRC) [35]. The ewes used in this study were owned by the Texas A&M Agricultural Experiment Station.

### 2.1. Experimental Design

**Figure 1:**
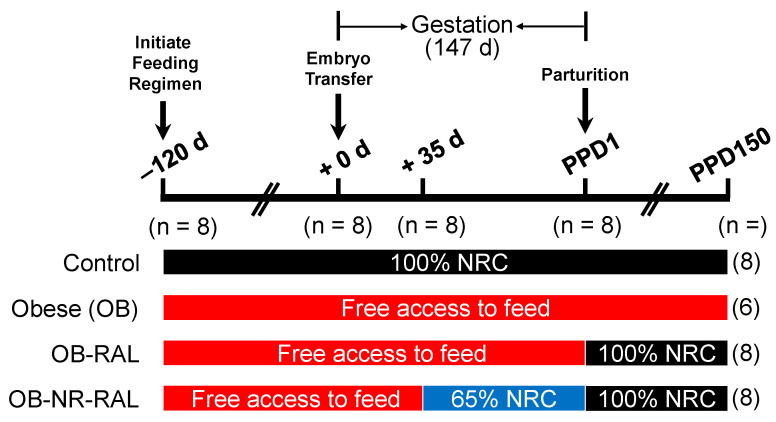
Experimental Design. At 120 days prior to estrus, Suffolk ewes were assigned randomly to either receive 100% of NRC nutrient requirements (*n* = 8) or have free access to feed (obesity induction; three groups of obese ewes; *n* = 8/group). After 120 days on the feeding regimens, each ewe received, by blastocyst transfer, a single blastocyst from a super-ovulated Suffolk ewe on day 6 post-estrus (i.e., +0 d). Thirty-five days after blastocyst transfer, one group of obese ewes was subject to restricted feeding (65% of feed intake for the 100% NRC-fed control group) until parturition, and the remaining two groups of obese ewes continued to have free access to feed throughout gestation. Following parturition, the control and one obese group continued to be fed 100% NRC nutrient requirements and to have free access to feed, respectively, whereas the other obese group and OB-NR groups were realimented to 100% NRC nutrient requirements (OB-RAL and OB-NR-RAL, respectively). In the OB group, two ewes died within 1 month (one ewe on PPD12 and another ewe on PPD30) after parturition. The numbers of ewes per treatment group at the different time points of the experiment are indicated in parentheses. Note that only 6 ewes remained in the OB group for subsequent measurements on PPD150. d, days; PPD, post-partum days.

The experimental design is illustrated in Figure 1. At 120 days prior to estrus, multiparous Suffolk ewes were assigned randomly to either receive 100% of NRC nutrient requirements (control group, *n* = 8) or have free access to feed (obesity induction; three groups of obese ewes; *n* = 8/group), as described by Satterfield [34]. The number of ewes per treatment group was based on the statistical power calculation as we described previously [36]. Ewes in the control group were maintained on 100% NRC feeding throughout the entire experimental period, whereas the OB group had free access to feed throughout the entire experimental period. Before pregnancy, the feed intake of ewes in the 100% NRC-fed group was 20.0 g feed/kg body weight per day, whereas ewes in the obesity-induction group consumed 170% of the feed intake for the 100% NRC group on a per-animal basis. After 120 days on the feeding regimens, 100% NRC-fed ewes (with an average BCS of 3.0) and all obese ewes (with an average BCS of 4.5) were synchronized into estrus and a single blastocyst from a super-ovulated Suffolk ewe with a normal BCS of 3.0 was transferred into the uterus on day 6 post-estrus to generate genetically similar fetuses in singleton pregnancies as we described previously [34,37]. Pregnancy was confirmed by ultrasound on day 28 of gestation.

Thirty-five days after embryo transfer, 8 obese ewes were subject to restricted feeding (NR; 65% of feed intake for the 100% NRC-fed control group) until parturition, and the remaining 16 obese ewes (8 ewes/group) continued to have free access to feed throughout gestation. During pregnancy, the feed intake of ewes in the 100% NRC-fed group was 22.7 g feed/kg body weight per day, whereas ewes in the OB group consumed 175% of the feed intake for the 100% NRC group on a per-animal basis. Following parturition, offspring were removed from their mothers and used for another study to assess postnatal growth and metabolism [38], the control group continued to be fed 100% NRC nutrient requirements, the OB group continued to have free access to feed, one obese group was realimented to 100% NRC nutrient requirements (OB-RAL), and the OB-NR ewes were realimented to 100% NRC nutrient requirements (OB-NR-RAL). During the 150-day post-partum period, the feed intake of ewes in the 100% NRC-fed groups was 26.1 g feed/kg body weight per day, whereas the feed intake of ewes in the OB group was 172% of the feed intake for the 100% NRC-fed group on a per-animal basis. In the OB group, two ewes died within 1 month (one ewe on PPD12 and another ewe on PPD30) after parturition, and therefore only 6 ewes remained in this group for subsequent measurements on PPD150. During pregnancy and after parturition, all ewes were individually housed, fed daily, and weighed weekly, whereas feed intakes for the 100% NRC-fed control ewes, the OB-RAL ewes, and the OB-NR-RAL ewes were adjusted based on body frame size.

### 2.2. Glucose Tolerance Test (GTT)

On postpartum day 1 (PPD1) and postpartum day 150 (PPD150), following a 12 h period of food deprivation, ewes were subjected to a glucose tolerance test by the intravenous infusion of a 50% glucose solution (0.25 mg/kg body weight) [39]. Blood samples were obtained from the jugular vein in 3 mL EDTA-K_2_ tubes (BD Vacutainer) at 0, 5, 10, 15, 30, 60, 120, and 180 min after the bolus injection of glucose solution. Blood samples were immediately centrifuged at 3500× *g* for 5 min to obtain plasma, which was aliquoted into 1.5 mL tubes and stored at −20 °C until analyzed.

### 2.3. Biochemical Analyses of Plasma from Ewes

Concentrations of leptin in plasma were determined as described by Delavaud et al. [40]. Concentrations of insulin in plasma were measured by EIA (Catalog # 80-INSOV-E01, ALPCO Diagnostics, Salem, NH, USA) according to the manufacturer’s recommendations. Non-esterified fatty acids (NEFA) were determined using a commercial colorimetric assay (Wako Chemicals, Richmond, VA, USA). Amino acids, glucose, ammonia, and urea in plasma were analyzed using HPLC and enzymatic methods, as we described previously [41].

### 2.4. Statistical Analysis

Values are least square means and pooled SE. Data were analyzed by ANOVA using the General Linear Model procedures of the Statistical Analysis System (SAS/STAT Software, SAS Institute, Inc., Cary, NC, USA). Leptin was analyzed using 1-way ANOVA, whereas all others were analyzed using 2-way ANOVA, with diet and time as the main effects. Values of *p* ≤ 0.05 were taken to indicate statistical significance.

## 3. Results

### 3.1. Body Weights of Ewes on PPD1 and PPD150

On PPD1, body weights of control, obese, and OB-NR ewes were 80.0, 116, and 86.4 kg (pooled SEM = 3.75 kg), respectively. Obese ewes were 45% and 34% heavier (*p* < 0.01) than control and OB-NR ewes, respectively. On PPD150, body weights of control, obese, OB-RAL, and OB-NR-RAL ewes were 70, 117, 98, and 80 kg (pooled SEM = 4.1 kg), respectively (*p* < 0.0001). Body weights of ewes differed (*p* < 0.05) among the obese, OB-RAL, and OB-NR-RAL groups, whereas no difference was detected between the control and OB-NR-RAL groups. During the course of the study, two ewes died.

### 3.2. Concentrations of Leptin in Plasma

On PPD1, concentrations of leptin in plasma were greater in both OB-NR (*p* < 0.05) and obese (*p* < 0.01) ewes than in control-fed ewes (Table 1). On PPD150, concentrations of leptin in plasma from OB-RAL and obese ewes were greater (*p* < 0.05 and *p* < 0.01, respectively) than those for the control ewes, but values for OB-NR-RAL and control ewes did not differ (Table 1). Additionally, obese ewes had a 79% increase (*p* < 0.05) in concentrations of leptin in plasma over the values for OB-RAL ewes.

### 3.3. Concentrations of Insulin in Plasma

On PPD1, concentrations of insulin in plasma differed (*p* < 0.01) due to time and treatment after intravenous administration of the glucose solution (Table 2). Baseline concentrations of insulin in plasma did not differ among treatment groups. Peak concentrations of insulin occurred at 10 min for control-fed and obese ewes and at 30 min for OB-NR ewes. Circulating levels of insulin returned to baseline values by 30 min for control-fed ewes and by 120 min for obese and OB-NR ewes.

On PPD150, there were effects of both treatment and time on concentrations of insulin in plasma (*p* < 0.01), but their interaction was not significant (Table 2). Baseline concentrations of insulin in plasma did not differ among treatment groups. In both control-fed and OB-RAL ewes, concentrations of insulin in plasma peaked at 10 min. However, circulating levels of insulin returned to baseline by 60 min for control-fed ewes and 120 min for OB-RAL ewes. In contrast, concentrations of insulin in the plasma of obese and OB-NR-RAL ewes peaked at 30 min and returned to basal levels by 60 and 120 min, respectively. OB-RAL ewes had higher (*p* < 0.01) concentrations of insulin in plasma at 10 min compared with all other groups and at 30 min compared with control-fed and OB-NR-RAL ewes. Concentrations of insulin in the plasma of obese ewes remained higher (*p* < 0.01) than those for all other groups at 120 min post-administration of the glucose solution.

### 3.4. Concentrations of Ammonia, Urea, and Non-Esterified Fatty Acids in Plasma

On PPD1 and PPD150, there were no effects of time on either ammonia or urea levels in plasma; therefore, data were pooled across time for analysis by ANOVA. On PPD1, concentrations of ammonia and urea in plasma were not different among the three groups of ewes (Control, Obese, and OB-NR), but circulating levels of NEFA were affected (*p* < 0.05) by dietary treatment (Table 1). Obese ewes had higher (*p* < 0.05) levels of NEFA in plasma than control-fed ewes, and OB-NR ewes had intermediate levels of circulating NEFA.

On PPD150, concentrations of ammonia and urea in plasma were affected by dietary treatment (Table 1). Obese and OB-NR-RAL ewes had lower (*p* < 0.05) concentrations of ammonia in plasma than control-fed ewes, but values for OB-RAL ewes did not differ from other treatments. Obese ewes had higher (*p* < 0.05) concentrations of urea in plasma than control, OB-NR-RAL, and OB-RAL ewes. The effect of treatment was detected (*p* < 0.01) for concentrations of NEFA in plasma (Table 1). In particular, obese ewes had higher (*p* < 0.01) concentrations of NEFA in plasma than OB-RAL, OB-NR-RAL, and control-fed ewes. Further, OB-RAL ewes had higher (*p* < 0.05) levels of NEFA than OB-NR-RAL and control-fed ewes. Concentrations of NEFA in plasma did not differ between OB-NR-RAL and control-fed ewes.

### 3.5. Concentrations of Glucose in Plasma

Whole-body utilization of glucose, indicated by GTT results, was affected by dietary treatments on both PPD1 and PPD150 (Table 3). On PPD1, basal concentrations of glucose in plasma did not differ among the three treatment groups. In response to the intravenous bolus of glucose, concentrations of glucose in plasma increased to peak values at 5 min post-administration for control-fed and OB-NR ewes and at 10 min for obese ewes. Between 10 and 30 min post administration of glucose, both OB-NR and obese ewes had greater (*p* < 0.05) concentrations of glucose in plasma than control-fed ewes, with the exception of obese ewes at 15 min. Circulating levels of glucose returned to baseline values by 60 min for control-fed ewes, but not until 120 min for both OB-NR and obese ewes. The area under the curve (AUC) and half-life (T_1/2_) were greater (*p* < 0.05), but clearance rate (CL) was lower (*p* < 0.05) for obese compared to control-fed ewes (Table 4). Maximum concentrations (C_max_) of glucose in plasma did not differ among treatment groups (Table 4).

On PPD150, basal levels of glucose in plasma did not differ among the four groups of ewes, but peak concentrations of glucose were at 5 min post-administration of glucose for all treatment groups (Table 3). Concentrations of glucose were higher (*p* < 0.05) in obese, OB-RAL, and OB-NR-RAL ewes than in control-fed ewes. At 10 to 15 min post-administration, obese, OB-RAL, and OB-NR-RAL ewes had higher (*p* < 0.05) concentrations of glucose than control-fed ewes. At 30 min, OB-NR-RAL ewes did not differ from control-fed ewes in concentrations of glucose in plasma, and at 60 min, neither realimented group differed from control-fed ewes. At 180 min, obese ewes still had higher (*p* < 0.001) concentrations of glucose in plasma than the control group. Concentrations of glucose returned to baseline concentrations by 60 min in control-fed ewes, 120 min in OB-NR-RAL and OB-RAL ewes, and 180 min in obese ewes (Table 3).

Glucose kinetics were affected by dietary treatments (Table 4). Area under the curve (AUC) for glucose did not differ among control-fed, OB-NR-RAL, and OB-RAL ewes, but obese ewes had greater (*p* < 0.05) values than all other treatment groups. Further, the half-life (T_1/2_) of glucose in plasma was longer (*p* < 0.05) in obese ewes than in all other treatment groups. In contrast, maximum concentrations (C_max_) were greater (*p* < 0.001) in obese ewes than in control-fed and OB-NR-RAL ewes. Finally, the clearance rate (CL) of glucose in plasma was lower (*p* < 0.05) in obese compared with control-fed ewes.

### 3.6. Concentrations of Amino Acids in Plasma

Concentrations of total amino acids in plasma were unaffected by diet or time on PPD1 (Table 5), but there were effects of treatment on specific amino acids. Concentrations of histidine increased (*p* < 0.05) in both obese and OB-NR compared with the control-fed ewes, while aspartate and citrulline decreased (*p* < 0.05 and *p* < 0.01, respectively). Concentrations of serine, glutamine, and glycine were greater (*p* < 0.01) in OB-NR ewes, while ornithine levels were lower (*p* < 0.05). Circulating levels of all branched-chain amino acids (BCAAs; isoleucine, leucine, and valine) and cysteine were also higher (*p* < 0.05) in obese ewes compared to ewes in the other treatment groups. Time after administration of the glucose tolerance test also had an effect on some amino acids, as concentrations of isoleucine, phenylalanine, tryptophan, tyrosine, and valine (control-fed and obese ewes) decreased over time (*p* < 0.05). Only proline increased (*p* < 0.05) after administration of the bolus of glucose. Concentrations of all other amino acids remained unchanged by either diet or time. Of note, methionine levels did not differ among treatments or across time, except for control-fed ewes, in which values were higher at 120 min (*p* < 0.01).

On PPD150, concentrations of most amino acids in plasma were affected (*p* < 0.05) by either diet or time (Table 6). Concentrations of alanine, glutamate, tyrosine, and tryptophan in plasma were higher in obese ewes with or without weight loss compared with control-fed ewes (*p* < 0.01). Concentrations of glycine in plasma were greater in both realimented groups (OB-NR-RAL and OB-RAL) (*p* < 0.05), but not in obese ewes. Circulating levels of glutamine (*p* < 0.05) and serine (*p* < 0.01) were greater in OB-NR-RAL ewes compared to control ewes. Concentrations of taurine, all BCAAs, cysteine, and phenylalanine were higher (*p* < 0.05) in the plasma of obese than control-fed ewes. Concentrations of glutamine increased (*p* < 0.05) over time, while alanine, β-alanine, cysteine, glutamine, leucine, phenylalanine, histidine, isoleucine, lysine, methionine, ornithine, taurine, threonine, tyrosine, valine, and total amino acids in plasma decreased (*p* < 0.05). Proline concentrations were initially lower in obese ewes, but by 120 min after the glucose tolerance test, these ewes had greater levels of proline, while all other groups exhibited a decrease (*p* < 0.05, treatment × time). Concentrations of arginine, asparagine, aspartate, and citrulline did not change (*p* > 0.05) in response to diet or time.

## 4. Discussion

Obesity during gestation occurs in farm animals such as ewes [4,5,6,7,8] and is a common and growing problem around the world [9,13,14,15]. Although the literature is vast on the implications of maternal obesity on the fetus during pregnancy and offspring after birth [17,22,23,24,25,26], very little is known about either short-term or long-term metabolic impacts of maternal obesity on glucose kinetics, amino acid availability, and concentrations of ammonia and urea in mothers. The present study involved an obese ewe model to elucidate some of the consequences of this growing problem in obese pregnant women. Obesity was evident in ewes before pregnancy to mimic obese women who unknowingly become pregnant. Our results indicate that gestational obesity impaired insulin sensitivity on both PPD1 and PPD150, which resulted in a vast amount of downstream adverse effects, including reduced utilization of glucose (Table 3 and Table 4) as well as altered metabolism of fatty acids (Table 1) and amino acids (Table 5 and Table 6).

Obesity can impair the oxidation of fatty acids and glucose in animals [2,42,43,44,45]. Such an effect, coupled with increased intake of energy substrates (including lipids and carbohydrates) from the diet, causes high levels of NEFA (Table 1) in plasma and particularly glucose in response to its administration (Table 3). High concentrations of long-chain saturated fatty acids and glucose are known to result in insulin resistance in skeletal muscle and other tissues of obese subjects, as reported for type-2 diabetic patients [46,47,48,49]. An important finding of this work is that reducing body weight (mainly white fat) during pregnancy or after parturition improved the metabolic profiles of the ewes. Specifically, ewes assigned to obesity management treatments exhibited improvements in concentrations of leptin, NEFA, insulin, and urea in plasma, as well as glucose metabolism, in comparison to the obese ewes on both PPD1 and PPD150 (Table 1 and Table 2). Because the ewes were followed for 5 months, the long-term effects of both maternal obesity and the management of this condition could be evaluated. To the best of our knowledge, this is the first report of effective and safe intervention methods for ameliorating metabolic syndrome in ewes with gestational obesity.

Obesity is known to blunt anabolic responses to dietary protein intake [50,51,52,53,54] and the expression of genes involved in amino acid metabolism [55] in non-pregnant adult animals. However, little is known about the effects of maternal obesity on this biochemical event in the mothers either immediately or in the long term following parturition. As an initial step to address this question, we determined concentrations of amino acids in ewes on PPD1 and PPD150. As in non-pregnant rats, maternal obesity altered the amino acid profiles in plasma on both days (Table 5 and Table 6). Of particular note, circulating levels of all BCAAs were greater in obese than control-fed ewes. This suggests impaired mitochondrial function in skeletal muscle, the major site for initiation of BCAA degradation in mammals, including humans [56,57,58] and sheep [59,60,61]. In contrast, concentrations of serine and citrulline were reduced in the plasma of obese ewes on PPD1 (Table 5), possibly due to reduced synthesis from glycine and glutamine, respectively. In support of this view, concentrations of glycine and glutamine were higher in obese than control-fed ewes on PPD1 (Table 5). Altered metabolism of serine and impaired synthesis of citrulline likely result in adverse metabolic effects because of the following two reasons. First, serine is a major component of one-carbon unit metabolism that is essential to cell growth and differentiation [62,63]. Second, citrulline is the immediate substrate for intracellular synthesis of arginine [64], which is the precursor of nitric oxide (a major vasodilator, a key angiogenic factor, and a gaseous signaling molecule) [65,66].

Concentrations of ammonia and urea in plasma can be a good indicator of altered metabolism of protein and amino acids in animals [67], including sheep [68,69] and humans [70]. In keeping with this notion, circulating levels of urea were elevated in obese ewes, but reduced in obese ewes that lost weight either during pregnancy or after parturition (Table 1). Likewise, a reduction in concentrations of ammonia in the plasma of ewes at PPD150 (Table 1) may result from an increase in its use for the formation of glutamate (Table 6) by glutamate dehydrogenase [67]. Due to insulin resistance, protein synthesis is reduced, but protein degradation is increased in skeletal muscle of obese subjects [71,72], resulting in increased amounts of amino acids for oxidation and urea production [42,73]. This metabolic problem is diminished in obese mothers when their whole-body insulin action is enhanced through weight management, as indicated by reduced concentrations of urea in plasma (Table 1).

Obesity, along with excess gestational weight gain, can negatively impact maternal health [1,2,3]. Kiel et al. [74] reported that 46% of obese women gained more than 11.3 kg throughout gestation. Another study found that more than 70% of obese women gained more than the recommended amount of weight for obese pregnant women, and 21% gained more than 16 kg, which would be considered excessive even for women of normal pre-pregnancy weight [75]. Optimal weight management for obese women during pregnancy remains to be established [21]. In this regard, the results of this animal study may have important implications for the management of obese pregnant women. Sheep have similar metabolic profiles to humans, and so the findings obtained in this study should be easily translatable to conditions in humans [76,77,78]. Furthermore, the addition of obesity management treatments either during pregnancy or after parturition provides insight into how much the maternal condition can be improved when weight loss is induced, a critical subject area for future research involving maternal obesity [79,80,81]. Reducing the food intake of obese ewes to 65% of NRC nutrient requirements resulted in desirable weight loss (primarily white fat) [the present study]. Furthermore, our results indicate that initiation of weight loss even after parturition was highly beneficial for improving the metabolic profile in obese mothers (Table 1, Table 2, Table 3, Table 4, Table 5 and Table 6). These results can be helpful in recommending weight loss for obese pregnant women either during pregnancy or postpartum in an effort to improve long-term metabolic profiles of the mother.

## 5. Conclusions

Maternal obesity during gestation led to higher concentrations of leptin and NEFA in plasma, impaired glucose utilization, and an altered amino acid profile. Importantly, obesity management during gestation ameliorated these negative effects. Further, continued obesity for long-term periods after parturition can exacerbate the problem of high leptin and NEFA levels, which can further impair the actions of insulin, as well as the metabolism of amino acids, lipids, and glucose in mothers. Additionally, obesity management beginning immediately after parturition greatly improved maternal metabolic conditions. These new findings greatly enhance the base of knowledge on the effects of maternal obesity on the mother and outcomes of dietary interventions for successful management of obesity in women during gestation and during the postpartum period.

## Figures and Tables

**Table 1:** Concentrations of leptin, ammonia, urea, and non-esterified fatty acids (NEFA) in plasma of ewes on PPD1 and PPD150 ^1^.

Group	*n*	Leptin	Ammonia	Urea	NEFA
		µg/L	µmol/L	mmol/L	mmol/L
PPD1					
Control	8	4.2 ^c^	126	5.0	0.36 ^c^
Obese	16	19.4 ^a^	156	6.6	0.63 ^a^
OB-NR	8	8.9 ^b^	141	5.6	0.49 ^b^
SEM		1.5	7.2	0.35	0.03
*p*-Value		<0.01	0.25	0.21	<0.05
PPD150					
Control	8	4.7 ^c^	142 ^a^	4.3 ^b^	0.20 ^c^
Obese	6	26.2 ^a^	79 ^b^	5.6 ^a^	0.57 ^a^
OB-RAL	8	14.6 ^b^	109 ^a,b^	4.2 ^b^	0.38 ^b^
OB-NR-RAL	8	8.6 ^b,c^	104 ^b^	4.6 ^b^	0.23 ^c^
SEM		1.4	6.0	0.13	0.02
*p*-Value		<0.01	<0.01	<0.01	<0.01

^1^ Values are means with pooled SEM. Means within a column without a common letter differ, *p* < 0.05. Control, 100% of National Research Council-recommended nutrient requirements; NR, nutrient restricted; OB, obese; PPD, post-partum days; RAL, realimented to 100% of National Research Council-recommended nutrient requirements.

**Table 2:** Concentrations of insulin in the plasma of ewes on PPD1 and PPD150 at 0–120 min after intravenous administration of glucose ^1^.

Group	*n*	Time After IV Administration of Glucose (min)					
		0	5	10	30	60	120
ng/mL							
PPD1							
Control	8	0.8	1.8 ^a^	2.2 ^a^	1.6 ^a^	1.9 ^a^	1.1 ^a^
Obese	16	0.6	1.6 ^a^	2.4 ^a^	1.8 ^a^	1.8 ^a^	0.7 ^ab^
OB-NR	8	0.4	1.1 ^b^	1.0 ^b^	1.1 ^b^	1.2 ^b^	0.6 ^b^
SEM		0.02	0.02	0.03	0.03	0.02	0.02
*p*-Value		0.48	<0.05	<0.01	<0.05	<0.05	<0.05
PPD150							
Control	8	0.3 ^c^	1.2 ^b^	1.8 ^b^	1.7 ^b^	0.7 ^c^	0.3 ^b^
Obese	6	1.0 ^a^	2.1 ^b^	2.1 ^b^	2.1 ^b^	2.1 ^a^	1.7 ^a^
OB-RAL	8	0.7 ^a,b^	2.3 ^a^	3.1 ^a^	3.0 ^a^	1.7 ^a,b^	0.6 ^b^
OB-NR-RAL	8	0.5 ^b,c^	1.6 ^ab^	1.7 ^b^	2.1 ^b^	1.2 ^b,c^	0.3 ^b^
SEM		0.01	0.02	0.02	0.01	0.02	0.01
*p*-Value		<0.01	<0.01	<0.01	<0.01	<0.01	<0.01

^1^ Values are means with pooled SEM. Means within a column without a common letter differ, *p* < 0.05. Control, 100% of National Research Council-recommended nutrient requirements; iv, intravenous; NR, nutrient restricted; OB, obese; PPD, post-partum days; RAL, realimented to 100% of National Research Council-recommended nutrient requirements.

**Table 3:** Concentrations of glucose in the plasma of ewes on PPD1 and PPD150 at 0–180 min after intravenous administration of glucose ^1^.

Group	*n*	Time After IV Administration of Glucose (min)							
		0	5	10	15	30	60	120	180
		mmol/L							
PPD1									
Control	8	3.8	12.7	9.8 ^b^	9.7 ^b^	7.6 ^b^	5.9	3.9	3.0
Obese	16	3.5	13.2	13.7 ^a^	10.9 ^b^	9.9 ^a^	7.0	4.7	3.5
OB-NR	8	3.7	13.2	12.3 ^a^	13.4 ^a^	9.9 ^a^	7.6	4.4	3.4
SEM		0.03	0.03	0.03	0.04	0.03	0.04	0.03	0.03
*p*-Value		0.82	0.67	<0.01	<0.01	<0.01	0.16	0.53	0.69
PPD150									
Control	8	2.6	10.3 ^b^	9.1 ^b^	8.2 ^b^	5.9 ^c^	3.8 ^b^	2.5 ^b^	2.5 ^b^
Obese	6	3.6	12.6 ^a^	12.1 ^a^	11.3 ^a^	9.9 ^a^	9.3 ^a^	6.1 ^a^	4.6 ^a^
OB-RAL	8	3.1	12.4 ^a^	11.1 ^a^	10.3 ^a^	7.7 ^b^	5.2 ^b^	3.0 ^b^	2.6 ^b^
OB-NR-RAL	8	2.8	11.9 ^a^	10.9 ^a^	9.7 ^a^	7.2 ^b,c^	5.0 ^b^	3.7 ^b^	2.7 ^b^
SEM		0.02	0.02	0.02	0.02	0.02	0.02	0.02	0.02
*p*-Value		0.24	<0.05	<0.01	<0.01	<0.01	<0.01	<0.01	<0.01

^1^ Values are means with pooled SEM. Means within a column without a common letter differ, *p* < 0.05. Control, 100% of National Research Council-recommended nutrient requirements; Iv, intravenous; NR, nutrient restricted; OB, obese; PPD, post-partum days; RAL, realimented to 100% of National Research Council-recommended nutrient requirements.

**Table 4:** Kinetics of glucose in plasma of ewes on PPD1 and PPD150 after intravenous administration of glucose ^1^.

Group	*n*	AUC	CL	C_max_	T_1/2_
		(min·mL/mmol/L min mmol)/L(min·kg)			
PPD1					
Control	8	1548 ^b^	0.93 ^a^	9.3	111 ^b^
Obese	16	2021 ^a^	0.74 ^b^	9.5	148 ^a^
OB-NR	8	1479 ^b^	0.95 ^a^	9.9	100 ^b^
SEM		91	0.04	0.33	6
*p*-Value		<0.05	<0.05	0.70	<0.05
PPD150					
Control	8	1280 ^b^	1.63 ^a^	6.6 ^c^	104 ^b^
Obese	6	2312 ^a^	0.78 ^c^	9.5 ^a^	167 ^a^
OB-RAL	8	1235 ^b^	1.17 ^b^	8.3 ^a,b^	103 ^b^
OB-NR-RAL	8	1327 ^b^	1.04 ^b,c^	7.8 ^b^	116 ^b^
SEM		127	0.05	0.21	6
*p*-Value		<0.05	<0.01	<0.01	<0.05

^1^ Values are means with pooled SEM. Means within a column without a common letter differ, *p* < 0.05. AUC—area under the glucose concentration curve; CL—clearance rate; C_max_—maximum concentration; Control, 100% of National Research Council-recommended nutrient requirements; NR, nutrient restricted; OB, obese; PPD, post-partum days; RAL, realimented to 100% of National Research Council-recommended nutrient requirements; T_1/2_—half-life.

**Table 5:** Concentrations of amino acids in the plasma of ewes on PPD1 at 0 and 120 min after intravenous administration of glucose ^1^.

	0 min			120 min			*p*-Value			
	Control	Obese	OB-NR	Control	Obese	OB-NR	SEM	Diet	Time	Diet × Time
	µmol/L									
Ala	202	192	202	179	206	179	11	0.93	0.63	0.73
Arg	188	176	163	178	142	127	10	0.30	0.19	0.85
Asn	26	34	37	22	29	33	2	0.10	0.30	0.99
Asp	8 ^a^	5 ^ab^	5 ^ab^	6 ^a,b^	4 ^b^	4 ^b^	0.5	<0.05	0.18	0.98
β-Ala	20	19	27	26	22	24	2	0.49	0.50	0.55
Cit	255 ^a^	156 ^c^	218 ^a,b^	245 ^a,b^	141 ^c^	219 ^a,b^	11	<0.01	0.73	0.96
Cys	82 ^b^	169 ^a^	97 ^b^	72 ^b^	145 ^a^	91 ^b^	8	<0.01	0.42	0.89
Gln	207 ^b^	270 ^a^	302 ^a^	218 ^b^	227 ^b^	295 ^a^	8	<0.01	0.44	0.36
Glu	75	94	107	85	102	116	6	0.13	0.47	0.99
Gly	403 ^c^	473 ^b^	807 ^a^	512 ^b^	465 ^b^	886 ^a^	30	<0.01	0.36	0.71
His	42 ^b^	62 ^a^	77 ^a^	43 ^b^	62 ^a^	71 ^a^	4	<0.05	0.86	0.92
Ile	60 ^b^	95 ^a^	64 ^b^	50 ^b^	61 ^b^	62 ^b^	4	<0.05	<0.05	0.19
Leu	77 ^b^	159 ^a^	84 ^b^	74 ^b^	103 ^b^	93 ^b^	6	<0.01	0.15	0.06
Lys	94	124	92	106	112	91	7	0.14	0.64	0.47
Met	19 ^b^	24 ^b^	20 ^b^	46 ^a^	27 ^b^	23 ^b^	2	<0.05	<0.01	<0.01
Orn	99 ^a^	85 ^a^	60 ^b^	88 ^a^	64 ^b^	51 ^b^	6	<0.05	0.23	0.90
Phe	49 ^a,b^	61 ^a^	53 ^a,b^	43 ^b^	45 ^a,b^	47 ^a,b^	3	0.24	<0.05	0.73
Pro	87 ^c^	116 ^b,c^	96 ^c^	141 ^a,b^	167 ^a^	173 ^a^	7	0.22	<0.01	0.72
Ser	82 ^a^	52 ^b^	97 ^a^	50 ^b^	47 ^b^	100 ^a^	5	<0.01	0.29	0.38
Tau	46	61	68	52	75	52	5	0.12	0.64	0.66
Thr	70	87	74	74	102	96	5	0.09	0.34	0.54
Trp	32 ^a^	34 ^a^	23 ^b^	10 ^c^	24 ^b^	17 ^b,c^	2	0.22	<0.01	0.29
Tyr	66 ^a,b^	68 ^a^	62 ^a,b^	47 ^b^	53 ^a,b^	52 ^a,b^	3	0.80	<0.05	0.80
Val	151 ^b^	210 ^a^	107 ^c^	100 ^c^	128 ^b,c^	110 ^c^	9	<0.05	<0.05	0.18
Total	2357	2662	2681	2449	2425	2491	68	0.52	0.42	0.56

^1^ Values are means with pooled SEM. Means within a row without a common letter differ, *p* < 0.05. Control, 100% of National Research Council-recommended nutrient requirements; NR, nutrient restricted; OB, obese; PPD, post-partum days; RAL, realimented to 100% of National Research Council-recommended nutrient requirements.

**Table 6:** Concentrations of amino acids in the plasma of ewes on PPD150 at 0 and 120 min after intravenous administration of glucose ^1^.

	0 min				120 min				*p*-Value			
	Control	Obese	OB-RAL	OB-NR-RAL	Control	Obese	OB-RAL	OB-NR-RAL	SEM	Diet	Time	Diet × Time
	µmol/L											
Ala	107 ^c^	248 ^a^	164 ^b^	153 ^b^	93 ^c^	191 ^b^	110 ^c^	88 ^c^	5	<0.01	<0.01	0.28
Arg	169	227	176	213	199	165	160	169	7	0.56	0.13	0.16
Asn	26	27	28	35	30	28	23	29	1	0.14	0.36	0.23
Asp	3	4	4	4	4	5	4	6	0.3	0.16	0.06	0.46
β-Ala	48 ^a^	30 ^b,c^	36 ^a,b^	45 ^a^	20 ^c,d^	16 ^d^	11 ^d^	21 ^c,d^	3	0.45	<0.01	0.86
Cit	200	208	224	213	179	195	176	202	8	0.87	0.15	0.82
Cys	88 ^b,c^	135 ^a^	104 ^b^	92 ^b^	76 ^c^	103 ^b^	72 ^c^	63 ^c^	3	<0.01	<0.01	0.72
Gln	207 ^c^	208 ^c^	220 ^c^	270 ^a,b^	288 ^a,b^	294 ^a^	239 ^b^	311 ^a^	7	<0.05	<0.01	0.34
Glu	42 ^c^	74 ^a^	70 ^a,b^	68 ^a,b^	40 ^c^	58 ^abc^	46 ^c^	52 ^b,c^	0.4	<0.01	<0.01	0.40
Gly	311 ^d^	378 ^c,d^	470 ^a,b^	480 ^a,b^	427 ^a,b,c^	341 ^cd^	486 ^a,b^	506 ^a^	19	<0.05	0.43	0.56
His	110 ^a^	71 ^a^	100 ^a^	107 ^a^	47 ^b^	33 ^b^	37 ^b^	47 ^b^	5	0.26	<0.01	0.80
Ile	61 ^a,b^	71 ^a^	69 ^a^	57 ^a,b^	54 ^b^	65 ^ab^	48 ^b^	46 ^b^	2	<0.05	<0.01	0.26
Leu	86 ^a,b^	110 ^a^	87 ^a,b^	91 ^a,b^	62 ^b,c^	84 ^b^	46 ^c^	56 ^c^	3	<0.01	<0.01	0.68
Lys	78 ^a,b^	82 ^a,b^	87 ^a,b^	96 ^a^	74 ^a,b^	62 ^bc^	44 ^c^	57 ^c^	3	0.59	<0.01	0.15
Met	17 ^a^	19 ^a^	18 ^a^	19 ^a^	15 ^a,b^	16 ^ab^	12 ^b^	12 ^b^	0.6	0.35	<0.01	0.28
Orn	109 ^a,b^	117 ^a^	108 ^a,b^	93 ^a,b^	84 ^b,c^	62 ^c^	59 ^c^	65 ^c^	4	0.41	<0.01	0.46
Phe	45 ^c^	66 ^a^	57 ^a,b^	52 ^b,c^	30 ^d,e^	42 ^cd^	28 ^e^	28 ^e^	1	<0.01	<0.01	0.38
Pro	134 ^a^	71 ^c^	141 ^a^	131 ^a^	112 ^a,b^	139 ^a^	121 ^a,b^	96 ^b,c^	4	0.13	0.77	<0.01
Ser	48 ^c^	51 ^c^	74 ^b^	80 ^a^	43 ^c^	45 ^c^	46 ^c^	69 ^b^	3	<0.01	0.06	0.60
Tau	34 ^b,c^	64 ^a^	41 ^bc^	44 ^b^	31 ^c,d^	41 ^b,c^	23 ^d^	30 ^c,d^	2	<0.05	<0.01	0.46
Thr	125 ^a,b^	116 ^b,c^	142 ^a,b^	161 ^a^	90 ^c^	87 ^c^	98 ^c^	83 ^c^	9	0.81	<0.05	0.76
Trp	25 ^c^	41 ^a^	34 ^a,b^	33 ^b^	35 ^a,b^	40 ^a^	36 ^a,b^	30 ^b,c^	1	<0.01	0.48	0.21
Tyr	64 ^a,b^	69 ^a^	69 ^a^	72 ^a^	56 ^b^	75 ^a^	59 ^b^	61 ^b^	1	<0.01	<0.05	0.08
Val	140 ^a,b^	169 ^a^	116 ^b,c^	158 ^a^	102 ^c^	126 ^b^	87 ^c^	88 ^c^	5	<0.05	<0.01	0.45
Total	2228 ^a,b,c^	2474 ^a,b^	2542 ^a,b^	2617 ^a^	2174 ^b,c^	2302 ^a,b,c^	2039 ^c^	2199 ^b,c^	49	0.43	<0.01	0.35

^1^ Values are means with pooled SEM. Means within a row without a common letter differ, *p* < 0.05. Control, 100% of National Research Council-recommended nutrient requirements; NR, nutrient restricted; OB, obese; PPD, post-partum days; RAL, realimented to 100% of National Research Council-recommended nutrient requirements.

## Data Availability

All data are contained within this article.
